# Virus interference between H7N2 low pathogenic avian influenza virus and lentogenic Newcastle disease virus in experimental co-infections in chickens and turkeys

**DOI:** 10.1186/1297-9716-45-1

**Published:** 2014-01-06

**Authors:** Mar Costa-Hurtado, Claudio L Afonso, Patti J Miller, Erica Spackman, Darrell R Kapczynski, David E Swayne, Eric Shepherd, Diane Smith, Aniko Zsak, Mary Pantin-Jackwood

**Affiliations:** 1 Exotic and Emerging Avian Viral Diseases Unit, Southeast Poultry Research Laboratory, Agricultural Research Service, U.S. Department of Agriculture, Athens, GA, USA

## Abstract

Low pathogenicity avian influenza virus (LPAIV) and lentogenic Newcastle disease virus (*l*NDV) are commonly reported causes of respiratory disease in poultry worldwide with similar clinical and pathobiological presentation. Co-infections do occur but are not easily detected, and the impact of co-infections on pathobiology is unknown. In this study chickens and turkeys were infected with a *l*NDV vaccine strain (LaSota) and a H7N2 LPAIV (A/turkey/VA/SEP-67/2002) simultaneously or sequentially three days apart. No clinical signs were observed in chickens co-infected with the *l*NDV and LPAIV or in chickens infected with the viruses individually. However, the pattern of virus shed was different with co-infected chickens, which excreted lower titers of *l*NDV and LPAIV at 2 and 3 days post inoculation (dpi) and higher titers at subsequent time points. All turkeys inoculated with the LPAIV, whether or not they were exposed to *l*NDV, presented mild clinical signs. Co-infection effects were more pronounced in turkeys than in chickens with reduction in the number of birds shedding virus and in virus titers, especially when LPAIV was followed by *l*NDV. In conclusion, co-infection of chickens or turkeys with *l*NDV and LPAIV affected the replication dynamics of these viruses but did not affect clinical signs. The effect on virus replication was different depending on the species and on the time of infection. These results suggest that infection with a heterologous virus may result in temporary competition for cell receptors or competent cells for replication, most likely interferon-mediated, which decreases with time.

## Introduction

Low pathogenicity avian influenza virus (LPAIV) and lentogenic Newcastle disease virus (*l*NDV) are two of the most economically important viruses affecting poultry worldwide. They originate from their natural reservoirs, wild birds, with cross species transmission to domestic poultry producing subclinical infections and occasionally upper respiratory disease. Both, LPAIV and *l*NDV are single-stranded, negative-sense RNA viruses. AIV’s are type A Orthomyxoviruses and are classified as low pathogenicity (LP) and high pathogenicity (HP) viruses based on their virulence in chickens and the presence of multiple basic amino acids at cleavage site of the HA precursor protein [1]. NDVs, also known as avian Paramyxovirus 1, are members of the genus *Avulavirus* in the Paramyxoviridae family. NDV’s also vary in the type and severity of the disease they produce, and different pathotypes, based on virulence in chicken and the sequences surrounding the protease cleavage site of the fusion (F) protein, have been described in poultry: viscerotropic velogenic, neurotropic velogenic, mesogenic, lentogenic or respiratory, and asymptomatic [2]. Highly pathogenic avian influenza (HPAI) and Newcastle disease (ND), caused upon the infection of poultry with virulent (velogenic and mesogenic) strains of NDV, are diseases notifiable to the World Organization for Animal Health [3].

Select *l*NDV strains are commonly used as live vaccines in the commercial poultry industry to protect from virulent forms of Newcastle disease virus (*v*NDV), which causes high mortality. Experimental infections of specific pathogen-free (SPF) chickens with these *l*NDV vaccine strains cause little to no clinical disease [2]. However when these viruses infect poultry in the field they can decrease productivity by inducing a mild respiratory disease, particularly exacerbated when co-infected with other respiratory pathogens or in combination with environmental stressors. In many developed countries, *v*NDV is not endemic in poultry and prevention focuses on biosecurity and the vaccination of poultry with both live and inactivated *l*NDV vaccines [2], of which LaSota and B1 are the most commonly used strains worldwide.

Similarly to *l*NDV, LPAIVs produce subclinical infections in SPF chickens, but under commercial rearing conditions accompanied by secondary pathogens including viruses, environmental stress and/or immunosuppression, these viruses can also produce mild to moderate respiratory disease [4]. Outbreaks of LPAI occur periodically, and in certain countries LPAI is endemic such as H9N2 LPAI across North Africa, Middle East and Asia, and H5N2 LPAI in Central America [5]. However, most poultry are not routinely vaccinated against LPAI, and when vaccine is used, primarily LPAIV inactivated vaccines or vectored vaccines are utilized; no live LPAIV vaccines are licensed for use.

Co-infections of poultry with LPAIV and *l*NDV present a complicated clinical picture confusing the identification and diagnosis of both of these viruses [6,7], and unfortunately little is known on the interactions between these two viruses when simultaneously infecting poultry. Co-infection of poultry with more than one bacterial and/or viral agent is common and often results in increased clinical signs when compared to single agent infections [8-13]. Conversely, infection of a host with one virus may affect infection by a second virus, a phenomenon explained by the occurrence of viral interference, in which cells infected by a virus do not permit multiplication of a second virus [14]. Measurable differences may include changes in tissue permissiveness or tropism, viral replication, patterns of virus progeny production and release, latency, pathology including immunopathology, and immunological responses [15]. In addition, viral interference may be detrimental to detecting viruses in co-infected flocks since lower or undetectable virus titers might fail to give a complete diagnosis [16].

Exposure to *l*NDV, either as live vaccines or field strains, is nearly unavoidable for commercial and non-commercial poultry worldwide, and co-infections with LPAIV are likely to occur. Both viruses replicate in epithelial cells of the respiratory and intestinal tracts, where there are trypsin-like enzymes, likely competing for target cells or replicating in adjacent cells. Whether co-infections with LPAIV and *l*NDV will exacerbate clinical signs of disease in infected birds or produce viral interference, masking infections by one or other virus, is unknown. In this study we examined the effect of co-infections of chickens and turkeys with LaSota *l*NDV vaccine strain and a LPAIV (A/turkey/VA/SEP/67/02 H7N2) by inoculating the viruses simultaneously or sequentially and determining differences in pathogenesis (clinical signs, lesions), presence of the viruses in tissues, duration and titer of virus shedding and seroconversion to both viruses. Such a study design replicates field situations in countries free of virulent NDV, but with active NDV vaccination programs and where LPAI outbreaks periodically occur [2,5].

## Materials and methods

### Viruses

The following viruses were obtained from the Southeast Poultry Research Laboratory (SEPRL) virus repository: *l*NDV, APMV1/chicken/US (NJ)/LaSota/1946 (vaccine strain); and LPAIV, A/turkey/VA/SEP/67/2002 (H7N2). The viruses were propagated in embryonating chicken eggs (ECE) as previously described [17]. Allantoic fluid was diluted in brain heart infusion (BHI) medium (BD Bioscience, Sparks, MD, USA) in order to obtain an inoculum with 10^7^ 50% egg infectious dose (EID_50_) per bird in 0.1 mL. A sham inoculum was made using sterile allantoic fluid diluted 1:300 in brain heart infusion (BHI) medium. The experiment was performed in laboratory biosafety level-3 enhanced (BSL-3E) and animal BSL-3E facilities at the SEPRL, United States Department of Agriculture, Agricultural Research Service, and procedures were reviewed by the SEPRL institutional biosecurity committee.

### Birds

Four-week-old specific pathogen free (SPF) white leghorn chickens and three-week-old SPF small white Beltsville turkeys were obtained from the SEPRL in-house flocks. The birds were housed in self-contained isolation units (modified Horsfall isolators) that were ventilated under negative pressure with inlet and exhaust HEPA-filtered air and maintained under continuous lighting. Feed and water were provided *ad libitum*. Birds were cared for in accordance to an SEPRL’s Institutional Animal Care and Use Committee approved animal use protocol.

### Experimental design

Birds were separated into control groups and virus-inoculated groups (Table 1). All treatment groups contained 12 birds and were inoculated by the intraocular and intranasal (choanal cleft) routes. A dose of 10^7^ EID_50_ of each virus or sham inoculum was administered in 0.1 mL split between the eye and choana. The viruses were given alone, simultaneously (one virus given immediately after the other), or sequentially (the second virus 3 days after the first) (Table 1 and Additional file 1). The second time point was chosen based on our experience and previous studies showing high virus replication at 3 days after virus inoculation [18,19]. The birds were observed for signs of illness over a 14 day period. Body weights were taken at the time of virus exposure (day 0) and 3 days post inoculation (dpi). Oropharyngeal (OP) and cloacal (CL) swabs were collected from all birds from 1 to 4 dpi, and at 6, 8, and 10 dpi to assess virus shedding. Two birds from each group were euthanized at 3 dpi and tissues were collected in 10% neutral buffered formalin to evaluate microscopic lesions and the extent of virus replication in tissues as described previously [18,20]. At 14 dpi birds were bled for serology and euthanized by the intravenous (IV) administration of sodium pentobarbital (100 mg/kg body weight).

**Table 1:** Experimental design

**Species**	**Groups**		**Days of sampling**		
	**Day 0**	**Day 3**	**Weigh**	**Virus detection: OP and CL swabs**	**Serology**
**Chickens**	-	-	0, 3, 6	1, 6, 14	14
	*l*NDV	-	0, 3	1, 2, 3, 4, 6, 8, 10	14
	LPAIV	-	0, 3	1, 2, 3, 4, 6, 8, 10	14
	*l*NDV + LPAIV	-	0, 3	1, 2, 3, 4, 6, 8, 10	14
	*l*NDV	LPAIV	0, 3, 6	4, 5, 6, 7, 9, 11, 14*	14
	LPAI	*l*NDV	0, 3, 6	4, 5, 6, 7, 9, 11, 14*	14
**Turkeys**	-	-	0, 3, 6	1, 6, 14	14
	NDV	-	0, 3	1, 2, 3, 4, 6, 8, 10	14
	LPAI	-	0, 3	1, 2, 3, 4, 6, 8, 10	14
	*l*NDV + LPAIV	-	0, 3	1, 2, 3, 4, 6, 8, 10	14
	*l*NDV	LPAIV	0, 3, 6	4, 5, 6, 7, 9, 11, 14*	14
	LPAIV	*l*NDV	0, 3, 6	4, 5, 6, 7, 9, 11, 14*	14

*These time points correspond to 1, 2, 3, 4, 6, 8, 11 days after inoculation with the second virus.

### Quantitative real-time RT-PCR

Oropharyngeal and cloacal swabs were collected in 2 mL of BHI broth with a final concentration of gentamicin (200 μg/mL), penicillin G (2000 units/mL), and amphotericin B (4 μg/mL) and kept frozen at -70 °C until processed. RNA was extracted using the MagMax AI/ND RNA isolation kit (Ambion, Inc. Austin TX, USA). Quantitative real time RT-PCR (qRT-PCR) for AIV and Newcastle disease virus (NDV) detection was performed as previously described [21,22] with modifications. qRT-PCR reactions targeting the influenza virus M gene [23] and NDV M gene [24] were conducted using AgPath-ID one-step RT-PCR Kit (Ambion, Austin, TX, USA) and the ABI 7500 Fast Real-Time PCR system (Applied Biosystem, Calsbad, CA, USA). The RT step conditions for both primer sets were 10 min at 45 °C and 95 °C for 10 min. The cycling conditions for AIV were 45 cycles of 15 s, 95 °C; 45 s, 60 °C; and for NDV were 40 cycles of 10 s, 94 °C; 30 s, 56 °C; 10 s, 72 °C. The calculated qRT-PCR lower detection limit was for AIV was 10^0.5^EID_50_/mL and 10^1.6^EID_50_/mL for NDV. A standard curve for virus quantification was established with RNA extracted from dilutions of the same titrated stock of the challenge virus, and results also reported as EID_50_/mL equivalents.

### Serology

Hemagglutination inhibition (HI) assays were performed to quantify antibody responses to virus infection as previously described [3] with serum collected from birds at 14 dpi (11 dpi from the second virus in groups exposed to the viruses sequentially). Titers were calculated as the highest reciprocal serum dilution providing complete hemagglutination inhibition. Serum titers of 1:8 (2^3^) or lower were considered negative for antibodies against AIV or NDV.

### Statistical analyses

Data were analyzed using Prism v.5.01 software (GraphPad Software Inc. La Jolla, CA, USA) and values are expressed as the mean ± standard error of the mean (SEM). One-way ANOVA with Tukey post-test was used to analyze HI titers and body weights. The number of birds shedding virus were tested for statistical significance using Fisher’s exact test. Two-way ANOVA with Bonferroni multiple comparison analysis was used to evaluate virus titers in swabs. For statistical purposes, all qRT-PCR negative oropharyngeal and cloacal swabs were given a numeric value of 10^0.5^EID_50_/mL for AIV and 10^1.6^EID_50_/mL for NDV. All HI-negative serum was given a value of 3 log_2_. These values represent the lowest detectable level of virus and antibodies in these samples based on the methods used. Statistical significance was set at *p* < 0.05 unless otherwise stated.

## Results

### Clinical signs

None of the chickens inoculated with LPAIV or *l*NDV, individually or co-infected with both viruses, showed clinical signs. All turkeys exposed to LPAIV, regardless of *l*NDV exposure, presented mild clinical signs consisting of mild periocular edema, mild conjunctivitis, mild sinusitis, ruffled feathers, and mild lethargy (Additional file 2). These clinical signs were first observed at 3 dpi and lasted until 7 dpi. No differences in the severity of the clinical signs were observed between the groups inoculated with both LPAIV and *l*NDV and the group inoculated only with LPAIV. No clinical signs were observed in turkeys exposed to *l*NDV alone.

No significant differences in body weights were observed in virus-inoculated chickens and turkeys when compared to the controls (data not shown).

### Gross lesions, microscopic lesions and viral antigen staining in tissues

No gross lesions were observed in any of the birds necropsied at 3 dpi, except for the turkeys inoculated with LPAIV, which had mild conjunctivitis and sinusitis. The microscopic lesions observed were consistent with LPAIV and *l*NDV infection or non-specific inflammation typically observed with respiratory virus infection of poultry. Lesions present in tissues from LPAIV-infected turkeys included mild to moderate lymphoplasmacytic rhinitis, sinusitis and tracheitis. Mild lymphocytic rhinitis was observed in chickens.

NDV and AIV antigen staining was rare in tissues collected from LPAIV and *l*NDV-inoculated chickens and from the *l*NDV-inoculated turkeys and in both groups was confined to the nasal and trachea epithelial cells and infiltrating macrophages. More widespread AIV viral antigen staining was observed in tissues collected from turkeys inoculated with the LPAIV. Staining was present in the epithelial cells and infiltrating macrophages of the nasal turbinates, trachea, Harderian gland, and eyelid, and in the epithelial cells of the cloacal bursa (Additional file 2). No difference in the intensity or distribution of virus staining or in the severity of lesions was found between turkeys infected only with LPAIV and turkeys co-infected with LPAIV and *l*NDV.

### Viral shedding

#### *Number of birds shedding virus*

**Figure 1:**
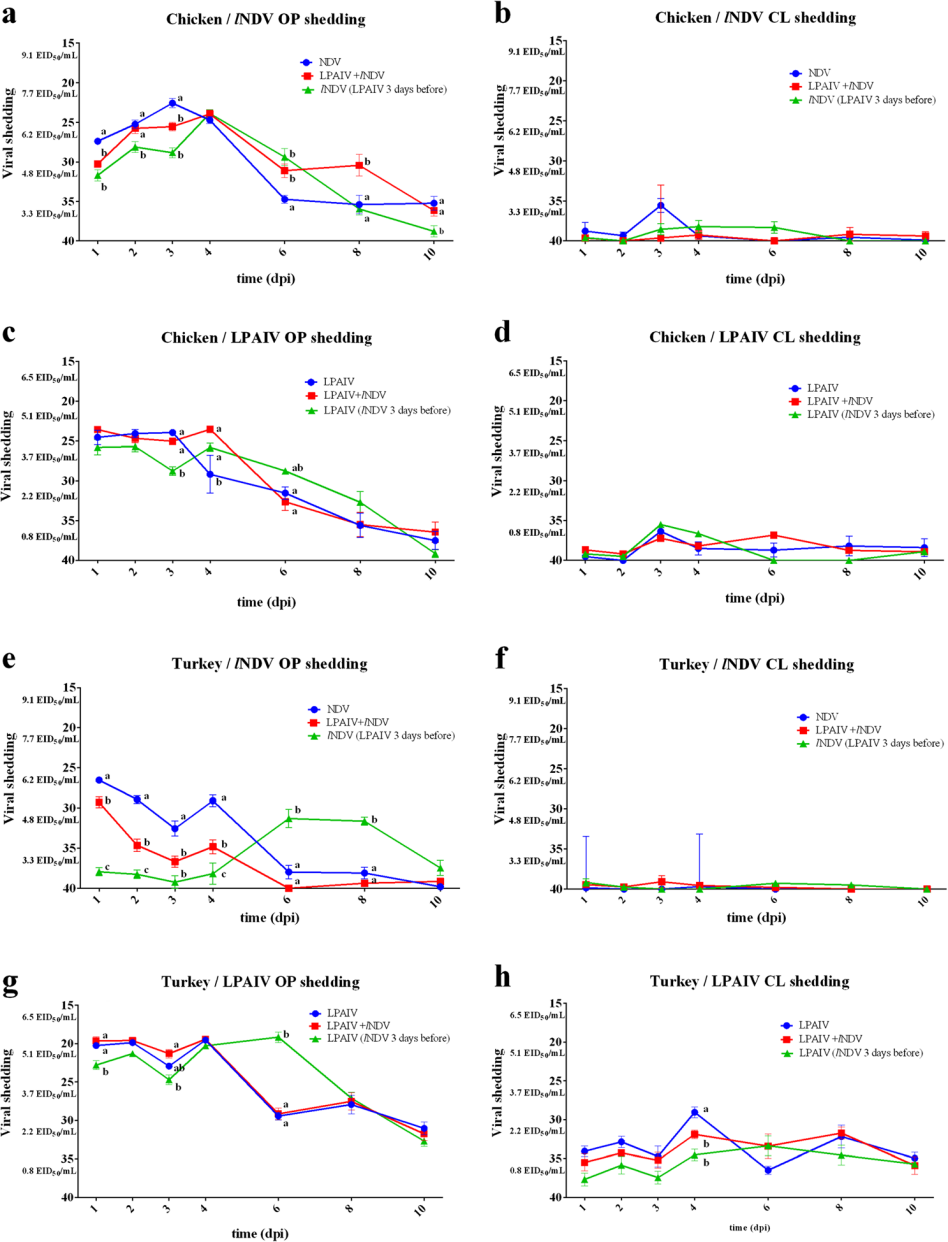
***l*NDV and LPAIV shedding in chickens and turkeys.** Each data point and their equivalent EID_50_/mL represents titers of *l*NDV and LPAIV detected in OP or CL swabs at different time points after inoculation, showing viral shedding of single infected birds (blue circles), simultaneously co-infected birds(red squares) and sequential infected birds (green triangles). Detection of *l*NDV in OP **(a)** and CL **(b)** swabs in chickens. Detection of LPAIV in OP **(c)** and CL **(d)** swabs in chickens. Detection of *l*NDV in OP **(e)** and CL **(f)** swabs in turkeys. Detection of LPAIV in OP **(g)** and CL **(h)** swabs in turkeys. In groups sequentially infected, the days post-inoculation (dpi) is based on the last virus given. Bars represent standard error of the mean.

Oral and cloacal viral shedding was examined by qRT-PCR and the results are shown in Tables 2 and 3 and Figure 1. All chickens and turkeys inoculated with *l*NDV and LPAIV became infected as was determined by the detection of the viruses in oropharyngeal and cloacal swabs (Tables 2 and 3). The total number of positive swabs and the duration of virus shedding varied between chickens and turkeys and virus exposure. For both chickens and turkeys, LPAIV viral shedding was mainly through the oropharyngeal (OP) route. A higher number of turkeys were shedding virus through this route at 8 and 10 dpi when compared with chickens. Most LPAIV-exposed turkeys had positive cloacal (CL) swabs until 10 dpi, contrary to the chickens in which the virus was detected inconsistently in most CL swabs.

**Table 2:** **Number of chickens positive for *l*NDV and LPAIV in oropharyngeal (OP) and cloacal (CL) swabs in single and co-infected groups**

**Virus detected**		**Group**		**Days post inoculation**							
		**Day 0**	**Day 3**	**1**	**2**	**3**	**4**	**6**	**8**	**10**	**Total**^**b**^
***l*** **NDV**	OP	*l*NDV		10/10^a^	10/10	10/10	10/10	10/10	7/10	9/10	66
		LPAIV		ND	ND	ND	ND	ND	ND	ND	-
		*l*NDV + LPAIV		10/10	10/10	10/10	10/10	10/10	10/10	10/10	70
		LPAIV	*l*NDV	10/10	10/10	10/10	10/10	10/10	10/10	4/10**	64
	CL	*l*NDV		2/10	2/10	2/10	2/10	0/10	1/10	1/10	10
		LPAIV		ND	ND	ND	ND	ND	ND	ND	-
		*l*NDV + LPAIV		2/10	0/10	1/10	2/10	0/10	0/10	2/10	7
		LPAIV	*l*NDV	3/10	0/10	4/10	4/10	3/10	0/10	0/10	14
**LPAIV**	OP	*l*NDV		ND	ND	ND	ND	ND	ND	ND	-
		LPAIV		10/10	10/10	10/10	10/10	10/10	8/10	4/10	62
		*l*NDV + LPAIV		10/10	10/10	10/10	10/10	9/10	9/10	6/10	64
		*l*NDV	LPAIV	10/10	10/10	10/10	10/10	10/10	10/10	2/10	62
	CL	*l*NDV		ND	ND	ND	ND	ND	ND	ND	
		LPAIV		2/10	1/10	10/10	3/10	3/10	3/10	2/10	24
		*l*NDV + LPAIV		4/10	1/10	5/10*	4/10	4/10	3/10	3/10	24
		*l*NDV	LPAIV	3/10	4/10	8/10	8/10*	2/10	1/10	1/10	27

In groups sequentially infected, the day post-inoculation (dpi) is based on the last virus given.

^a^Number of positive birds/total number of birds sampled at each time point.

^b^Total number of positive swabs.

ND, No data.

*Significant difference for number of positive chickens by qRT-PCR compared to single virus infected groups, (*, *P* < 0.05; **, *P* < 0.01).

**Table 3:** **Number of turkeys positive for *l*** **NDV and LPAIV in oropharyngeal (OP) and cloacal (CL) swabs in single and co-infected groups**

**Virus detected**	**Swab**	**Group**		**Days post inoculation**							
		**Day 0**	**Day 3**	**1**	**2**	**3**	**4**	**6**	**8**	**10**	**Total**^**b**^
***l*NDV**	OP	*l*NDV		10/10 ^a^	10/10	10/10	10/10	4/10	5/10	3/10	52
		LPAIV		ND	ND	ND	ND	ND	ND	ND	-
		*l*NDV + LPAIV		10/10	10/10	8/10	10/10	0/10	3/10	5/10	46
		LPAIV	*l*NDV	8/10	7/10	1/10***	2/10***	10/10	10/10*	6/10	44
	CL	*l*NDV		1/10	0/10	0/10	1/10	0/10	0/10	0/10	2
		LPAIV		ND	ND	ND	ND	ND	ND	ND	-
		*l*NDV + LPAIV		3/10	2/10	2/10	2/10	1/10	0/10	0/10	10
		LPAIV	*l*NDV	3/10	1/10	0/10	0/10	3/10	3/10	0/10	10
**LPAIV**	OP	*l*NDV		ND	ND	ND	ND	ND	ND	ND	-
		LPAIV		10/10	10/10	10/10	10/10	10/10	10/10	10/10	70
		*l*NDV + LPAIV		10/10	10/10	10/10	10/10	10/10	10/10	10/10	70
		*l*NDV	LPAIV	10/10	10/10	10/10	10/10	10/10	9/9	9/9	68
	CL	*l*NDV		ND	ND	ND	ND	ND	ND	ND	-
		LPAIV		10/10	10/10	10/10	10/10	10/10	10/10	10/10	70
		*l*NDV + LPAIV		9/10	10/10	9/10	10/10	9/10	10/10	9/10	66
		*l*NDV	LPAIV	5/10*	9/10	7/10	10/10	10/10	9/9	9/9	59

In groups sequentially infected, the day post-inoculation (dpi) is based on the last virus given.

^a^Number of positive birds/total number of birds sampled at each time point.

^b^Total number of positive swabs.

ND, No data.

*Significant difference for number of positive turkeys by qRT-PCR compared to single virus infected groups, (*, *P* < 0.05; *** *P* < 0.001).

The number of positive OP and CL swabs was higher in chickens than in turkeys after exposure to *l*NDV, as more chickens shed virus longer (Tables 2 and 3). As with LPAIV, the oropharynx was the main route of *l*NDV shedding in chickens. An effect on the number of birds shedding *l*NDV was found for turkeys co-infected with LPAIV: a lower number of *l*NDV positive OP swabs than in turkeys infected only with the *l*NDV; however, more co-infected birds had positive CL swabs. Significant differences in the number of co-infected chickens shedding LPAIV was found in CL swabs taken at 3 and 4 dpi (*p* < 0.05). A reduction in the number of positive birds shedding *l*NDV through OP route was found at 10 dpi in the group exposed to LPAIV 3 days prior to *l*NDV (*p* < 0.01) (Table 2). On the other hand, turkeys exposed to LPAIV 3 days prior to *l*NDV exposure had significant differences in the number of birds shedding *l*NDV through the OP route at 3 dpi and 4 dpi (*P* < 0.0001) when compared to birds infected with a single virus, switching to higher numbers of birds shedding *l*NDV at 8 dpi (Table 3).

#### *Titer of virus shed*

When comparing the amount of virus shed at different time points after inoculation, for the chickens the pattern of OP viral shed was different depending on virus exposure (Figure 1a-d). In regards to OP *l*NDV shed by chickens, differences were observed among the groups (Figure 1a). At early time points (1–3 dpi), co-infected birds presented lower amounts of viral shedding than birds receiving *l*NDV only. These differences were significant at 1 and 3 dpi when chickens co-infected with LPAIV and *l*NDV showed significantly lower viral titers compared to single *l*NDV infected birds. The viral titers shed during the single *l*NDV infection dropped at 6 dpi while in co-infected chickens this decrease in virus shedding was less evident. Significantly higher amounts of virus shed was seen at 6 dpi in co-infected chickens, and also at 8 dpi for sequentially infected birds, compared with birds only infected with *l*NDV. No significant differences between groups were found regarding *l*NDV cloacal shedding (Figure 1b).

Chickens inoculated with LPAIV only, LPAIV and *l*NDV simultaneously, and LPAIV three days after inoculated with *l*NDV shed similar amounts of LPAIV at 1 dpi and 2 dpi, but by 3 dpi the birds inoculated with LPAIV after previously receiving *l*NDV shed significantly less virus. Also, birds infected only with LPAIV showed a progressive decrease of viral shedding starting at 4 dpi. This drop in virus shedding did not occur in chickens co-infected simultaneously with LPAIV and *l*NDV until 6 dpi, when birds co-infected with AIV (*l*NDV given 3 days before) were shedding significantly more virus than birds only infected with LPAIV (Figure 1c). No significant differences among groups were found in the LPAIV cloacal viral shedding in chickens (Figure 1d).

A clear example of viral interference was found between LPAIV and *l*NDV with turkeys. The LPAIV viral shedding in turkeys infected with LPAIV only or simultaneously co-infected with *l*NDV was very similar for all time points. However, turkeys that received *l*NDV 3 days before showed significant lower levels of LPAIV shedding at 1 and 3 dpi. Strikingly, the time to peak of LPAIV shedding in these turkeys occurred at 6 dpi while LPAIV single infected birds or birds simultaneously infected with LPAIV and *l*NDV showed a steep reduction of LPAIV shedding at this time point (Figure 1g). These results show an effect of previous infection with *l*NDV on LPAIV shedding with a reduction of viral shedding at early time-points and subsequent delay in the peak of shedding. No significant differences in LPAIV cloacal shedding was found in turkeys except for a slight increase in turkeys infected only with LPAIV at 4 dpi (Figure 1h).

Interestingly, significant differences in OP *l*NDV shedding were found between the different groups of turkeys (Figure 1e). Birds infected with *l*NDV only, showed a decrease of viral shedding from 1 to 3 dpi, with an increase at 4 dpi, followed by a drop in virus shedding by 6 dpi. Turkeys co-infected simultaneously with LPAIV and *l*NDV presented a similar trend but with significantly lower levels of *l*NDV shedding compared to the single *l*NDV infected birds. Turkeys infected with *l*NDV after previous inoculation with LPAIV showed no or low *l*NDV shedding from 1–4 dpi, with an increase in titers at day 6 and 8 dpi, therefore indicating that *l*NDV replication is hampered by the presence of LPAIV. Most infected turkeys did not shed *l*NDV throughout cloacal route (Figure 1f).

No differences in *l*NDV and LPAIV shedding was observed in the groups that received the second virus sequentially when compared to the virus shedding patterns observed in the groups in which the viruses were given alone (results not shown).

### Serology

**Figure 2:**
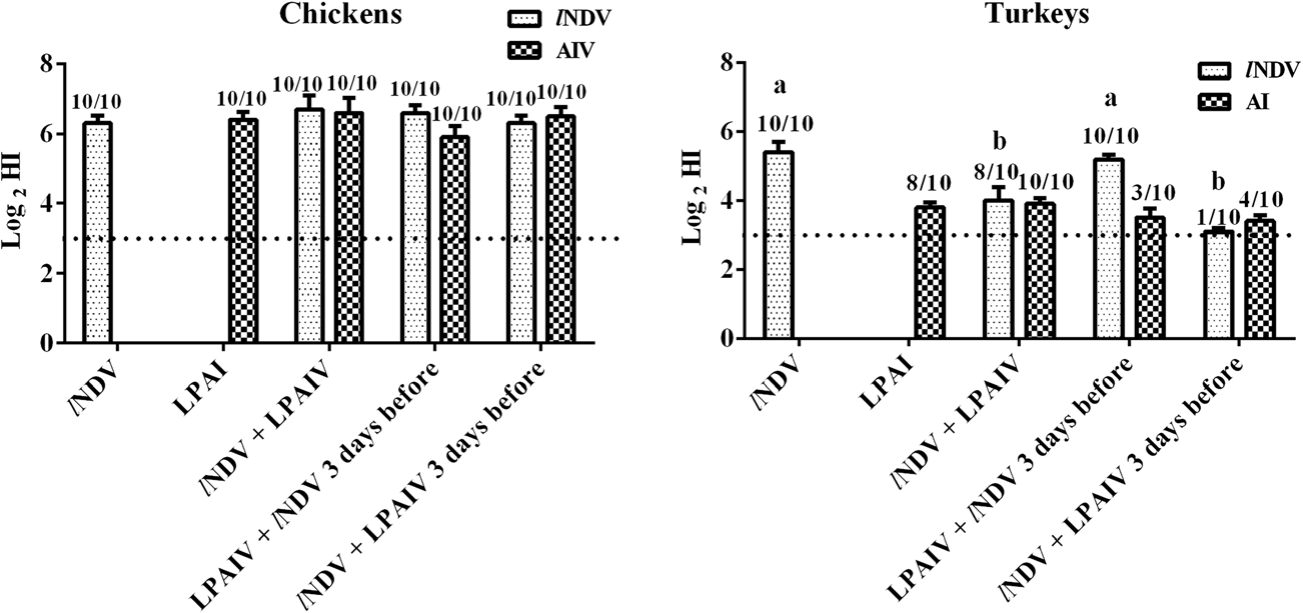
**Mean HI titers (log**_**2**_**) in chickens and turkeys.** Serum samples were taken at 11 and 14 days after infection with LPAIV, *l*NDV, or both. The number of birds with positive HI titers is shown (number of birds with titers ≥ the threshold of positivity/total animals per group). Different lowercase letters indicate significant differences in HI titers (*P* < 0.05) between groups. For statistical purposes, all HI-negative sera were given a value of 3 log2. Bars represent standard error of the mean.

HI assays were used to test for antibodies against LPAIV and *l*NDV (Figure 2). All chickens seroconverted to both LPAIV and *l*NDV, with no significant differences in titers among the treatment groups. Significant differences in *l*NDV HI titers were found between turkeys only infected with *l*NDV and turkeys simultaneously exposed to LPAIV and *l*NDV. Because serum samples were taken the same day for single, simultaneously and sequentially exposed birds (14 dpi for the first virus and 11 dpi for the second), not all groups are strictly comparable. Nevertheless, a clear difference was found in *l*NDV titers in turkeys exposed to LPAIV then *l*NDV 3 days later, indicating that the presence of LPAIV might be interfering with the production of antibodies against *l*NDV. However, a delay in the production of NDV HI titers because of a delay in viral replication cannot be ruled out (Figures 1e,g). The production of antibodies against LPAIV was not significantly different among groups and, in general, the titers were low. Nevertheless, eight or ten of ten turkeys inoculated only with LPAIV or simultaneously inoculated with *l*NDV seroconverted for LPAIV by 14 dpi, but only 3 or 4 of 10 turkeys had positive HI titers if previously infected by *l*NDV or infected 3 days after with *l*NDV.

## Discussion

Natural AIV/*l*NDV co-infections are expected to occur and have been reported in poultry [25-27], but the impact of such co-infections on several host responses including clinical outcome, viral shedding dynamics, seroconversion, and sites of virus replication in chickens and turkeys is unknown. Co-infections of AIV and NDV have been studied in vitro using cell cultures or chicken embryos, and interference between these viruses has been reported, with one virus inhibiting the growth of the other [7,28-32].

In contrast to in vitro or in ovo studies, in vivo experiments examine the overall effect of co-infections by incorporating the complexity of the whole organism, including different target cells and immune responses. In our co-infection studies, all chickens and turkeys became infected with *l*NDV and LPAIV, and a significant reduction in virus replication was observed when birds were co-infected versus single virus infected. However, this reduction in virus replication depended on the bird species, the virus, and the timing of inoculation. In spite of the differences in virus replication, co-infection of LPAIV and *l*NDV had no effect on the severity of clinical signs. Typically, chickens lack clinical signs in experimental infection with LPAIV or *l*NDV, which was corroborated by mild microscopic lesions and minimal virus antigen staining observed in respiratory tissues. Lack of clinical signs was also observed in turkeys infected with *l*NDV alone. However, all turkeys infected with LPAIV, co-infected or not, showed mild transient upper respiratory signs and moderate inflammation and necrosis in the epithelia of nasal cavity, trachea, Harderian gland, eyelid and cloacal bursa accompanied by LPAIV antigen staining. These results are consistent with previous reports suggesting LPAIV are more pathogenic for turkeys, which shed more virus from both OP and CL routes compared to chickens [19,33]. In addition, this H7N2 LPAIV strain is known to be turkey adapted, with a very low mean bird infectious dose required to produce infection [34]. Therefore, the host species is a factor that can influence the severity of clinical signs and amount of virus replication in such virus co-infections. In this study, an effect in the pattern of viral shedding was also found in the chickens, indicating that virus interference can occur, but to a lesser degree, as long as there is viral replication. Although we can only report what we found with these particular viruses, we do expect similar results with other viruses.

In this study, viral shedding patterns were clearly affected in chickens and turkeys exposed to *l*NDV and LPAIV simultaneously or sequentially. In chickens, although similar number of birds were shedding LPAIV virus, co-infection with *l*NDV delayed the peak of LPAIV OP shedding, with significantly less virus shed in birds previously inoculated with *l*NDV when measured at 3 dpi. Likewise, co-infection of chickens with LPAIV also affected *l*NDV OP virus shedding, with initial lower virus replication in co-infected birds than birds receiving only LPAIV, but higher and more prolonged virus shedding at later time points. Similar virus interference was also evident in the turkeys. Turkeys had higher LPAIV OP titers than the chickens, and all birds shed virus through the cloaca, regardless of co-infection with *l*NDV. The presence of high virus titers was associated with clinical signs and viral antigen staining in respiratory tissues of all turkeys infected with LPAIV. However, turkeys displayed initially lower levels of LPAIV when pre-infected with *l*NDV, and peaked later with higher titers. *l*NDV OP virus shedding was clearly affected by LPAIV replication, with fewer co-infected birds shedding *l*NDV and with significantly lower *l*NDV titers than turkeys infected with *l*NDV alone during the first four days. However, *l*NDV replication increased by 6 dpi in birds that received LPAIV three days earlier, probably because by then the effect of LPAIV replication had diminished. This suggests that infection with a heterologous virus may result in temporary competition for cell receptors or susceptible cells, resulting in decreased initial replication of the second virus; but as replication of the first virus declines, the second virus increases to fill the gap.

Viral interference is a phenomenon in which a cell infected by a virus does not permit multiplication of a second homologous or heterologous superinfectant virus [14]. Viral interference can be explained by different mechanisms including: competing by attachment interference therefore reducing or blocking of receptor sites for the superinfecting virus; competing intracellularly for replication host machinery; and virus-induced interferon interference [35]. *l*NDV and LPAIV replicate in cells where there are trypsin-like enzymes such as in the upper respiratory and intestinal epithelia [4,36] and might compete for the same target cells or replicate in adjacent cells. The LaSota virus, as a *l*NDV, binds through the HN glycoprotein to sialic acid-containing receptors on cell surface, as well as the HA glycoprotein does for LPAIV [37]. Replication of one virus might also be affected by previous replication in the same site of another virus that has already activated antiviral immune responses including immunomodulators or recruitment of immune cells. Although the LaSota *l*NDV strain is known to be a weak interferon inducer as part of their low virulent phenotype profile [38], local interferon production might still be able to interfere with LPAIV replication. In fact, previous studies in embryonating eggs showed that LaSota *l*NDV could suppress growth of a H9N2 LPAIV’s, if given prior to the LPAIV [28]. Influenza viruses also induce interferon [39,40], which could have been one mechanism by which the high LPAIV replication in the turkeys inhibited *l*NDV replication. Viral interference has also been suggested in other studies with influenza virus such as the pandemic H1N1 when it was shown that an increase in the proportion and number of rhinovirus diagnoses in humans occurred in parallel with the decrease of influenza diagnoses, suggesting that rhinoviruses inversely affected the spread of the pandemic H1N1 virus [41-43].

Experimental in vivo co-infections of NDV and LPAIV are scarce in chickens and turkeys. França et al. [44] performed co-infection in wild ducks with LPAIV and lentogenic NDV wild bird strains and observed differences in the pattern of virus shedding depending on the time of co-infection. A delay of the peak of LPAIV viral shedding was observed prior co-infection with NDV. The later the LPAIV inoculation was conducted in NDV infected animals, the less of a delay in the LPAIV OP shedding was found. The authors suggest that competition for replication sites and/or differences in fitness for replication may explain the effects of co-infection with lentogentic *l*NDV and LPAIV.

Other studies have examined co-infection of LPAIV and *l*NDV with other respiratory viruses of poultry. Research has shown that infectious bronchitis virus (IBV) interfered with the replication of *l*NDV [45-47]. However, IBV live vaccine increased the severity of H9N2 LPAIV infections [11,12], and it was suggested that IBV was a supplier of trypsin-like proteases therefore enhancing the reach of systemic sites by the virus. In our case, such an exacerbation would not occur, since neither the LaSota *l*NDV strain, nor the LPAIV can provide extra enzymatic activity to each other. In other studies, co-infection of turkeys with *l*NDV and another respiratory virus, avian pneumovirus (APV), induced more severe disease compared to turkeys infected with APV or NDV [48], and dual vaccination of turkeys with *l*NDV and hemorrhagic enteritis virus (HEV) live vaccines enhanced the pathologic response of the host [49]. In chickens, although the co-infection with LPAIV and *l*NDV interfered with viral replication as seen in the viral shedding patterns, the reduction in the humoral immune response was not observed, since all chickens seroconverted with similar antibody titers to LPAIV and NDV, regardless if they were co-infected or not. This is similar to what has been reported with experimental co-infections with IBV and live *l*NDV vaccines in broilers [47]. Although no significant effect of co-infection was observed on HI titers with these particular viruses, an effect might be seen in chickens infected with more virulent strains of NDV and AIV. On the other hand, not all turkeys seroconverted after infection. Turkeys are known to mount poor immune response to respiratory virus infections [50]. This was evident here, since lower antibody titers to LPAIV and *l*NDV were observed in turkeys compared to chickens. The lower number of turkeys showing LPAIV or *l*NDV antibody titers in the groups co-infected previously with either virus is also explained by the delay in viral replication found in those birds, not giving them the chance to seroconvert by 11 dpi. Interestingly, the production of antibodies against *l*NDV was impaired by the presence of LPAIV suggesting that *l*NDV had insufficient replication to trigger the humoral response despite the fact that they were administered the typical vaccination dose of *l*NDV LaSota strain. Thus, the humoral response of co-infected animals may not be affected in terms of antibody titers but may vary upon viral copies available to trigger the seroconversion.

Our co-infection study was performed under controlled conditions and using SPF birds in order to examine the specific interactions between the two viruses when given at high challenge doses. This might not be representative of what happens under field conditions where poultry are exposed to many viruses and other infectious and non-infectious disease agents. However, the results obtained underline the importance of co-infections which can either exacerbate clinical disease, or, like in our study, affect virus replication by lowering viral titers to under the levels of detection and affecting serological results, and in some cases increasing the time virus was shed which could favor prolonged transmission. The data also suggest that turkeys infected with LPAIV in the field may require either additional NDV booster vaccines or larger vaccine doses than normal to be able to mount a protective humoral immune response. In addition, exposure to lower challenge doses of these viruses in the field could also affect the results of co-infection. The effects of virus co-infection will most likely vary depending on how well adapted the viruses are to a specific bird species, on the virulence of the viruses involved, on the timing of co-infections, and on other concomitant infectious and environmental factors. Evaluating the infectious status in birds might be necessary when developing vaccination protocols using live attenuated vaccines.

The role of viral interference in the spread of AIV and NDV needs further examination as also the role of co-infections in terms of altering the severity of clinical signs and lesions. The identification of factors that influence co-infection interference or elements that favor a delay in infection of one virus at expense of another virus will provide new insights in the pathogenesis of these viruses, allowing a better design of diagnostic tools and improved vaccination to enhance control programs.

## Competing interests

The authors declare they have no competing interests.

## Authors’ contributions

MCH analyzed and interpreted the data and wrote the manuscript. CLA, PJM, ES, DK and DES were involved in the experimental design, interpretation of results and critically read the manuscript. ESh, DS and AZ prepared the viruses, helped conduct the animal studies, and processed and helped analyze the samples (HI and RRT-PCR). MPJ conceived the studies, coordinated the work described, conducted the animal experiment, the histopathology and immunohistochemistry, analyzed the data and edited the manuscript. All authors read and approved the final manuscript.

## Supplementary Material

Additional file 1 **Experimental design.** All treatment groups contained 12 birds. A dose of 10^7^ EID_50_ of the each virus or sham inoculum was administered in 0.1 mL split between the eye and choana. The viruses were given alone, simultaneously (one virus given immediately after the other), or sequentially (the second virus 3 days after the first). The birds were observed for signs of illness over a 14 day period. Body weights were taken at the time of virus exposure and 3 days post inoculation (dpi). Oropharyngeal (OP) and cloacal (CL) swabs were collected at different time points to assess virus shedding. Tissues were collected from 2 birds per group to evaluate microscopic lesions and the extent of virus replication in tissues. At 14 dpi birds were bled for serology and euthanized. Click here for file

Additional file 2 **Clinical signs and distribution of AI viral antigen in tissues collected from turkeys infected with LPAIV, 3 dpi. Infraorbital swelling and conjunctivitis in affected birds.** Viral antigen staining (in red) in the epithelium, desquamated cells and infiltrating inflammatory cells of the nasal turbinates, Harderian gland and eyelid, and in the bursa epithelium. Click here for file
