# Supplementary material for: Virus interference between H7N2 low pathogenic avian influenza virus and lentogenic Newcastle disease virus in experimental co-infections in chickens and turkeys
Source: Vet Res. 2014 Jan 6;45(1):1. doi: 10.1186/1297-9716-45-1 (PMC3890543; doi:10.1186/1297-9716-45-1)
Supplement: Additional file 1 — Experimental design. All treatment groups contained 12 birds. A dose of 107 EID50 of the each virus or sham inoculum was administered in 0.1 mL split between the eye and choana. The viruses were given alone, simultaneously (one virus given immediately after the other), or sequentially (the second virus 3 days after the first). The birds were observed for signs of illness over a 14 day period. Body weights were taken at the time of virus exposure and 3 days post inoculation (dpi). Oropharyngeal (OP) and cloacal (CL) swabs were collected at different time points to assess virus shedding. Tissues were collected from 2 birds per group to evaluate microscopic lesions and the extent of virus replication in tissues. At 14 dpi birds were bled for serology and euthanized. [file 1297-9716-45-1-S1.pptx]

## Slide 1
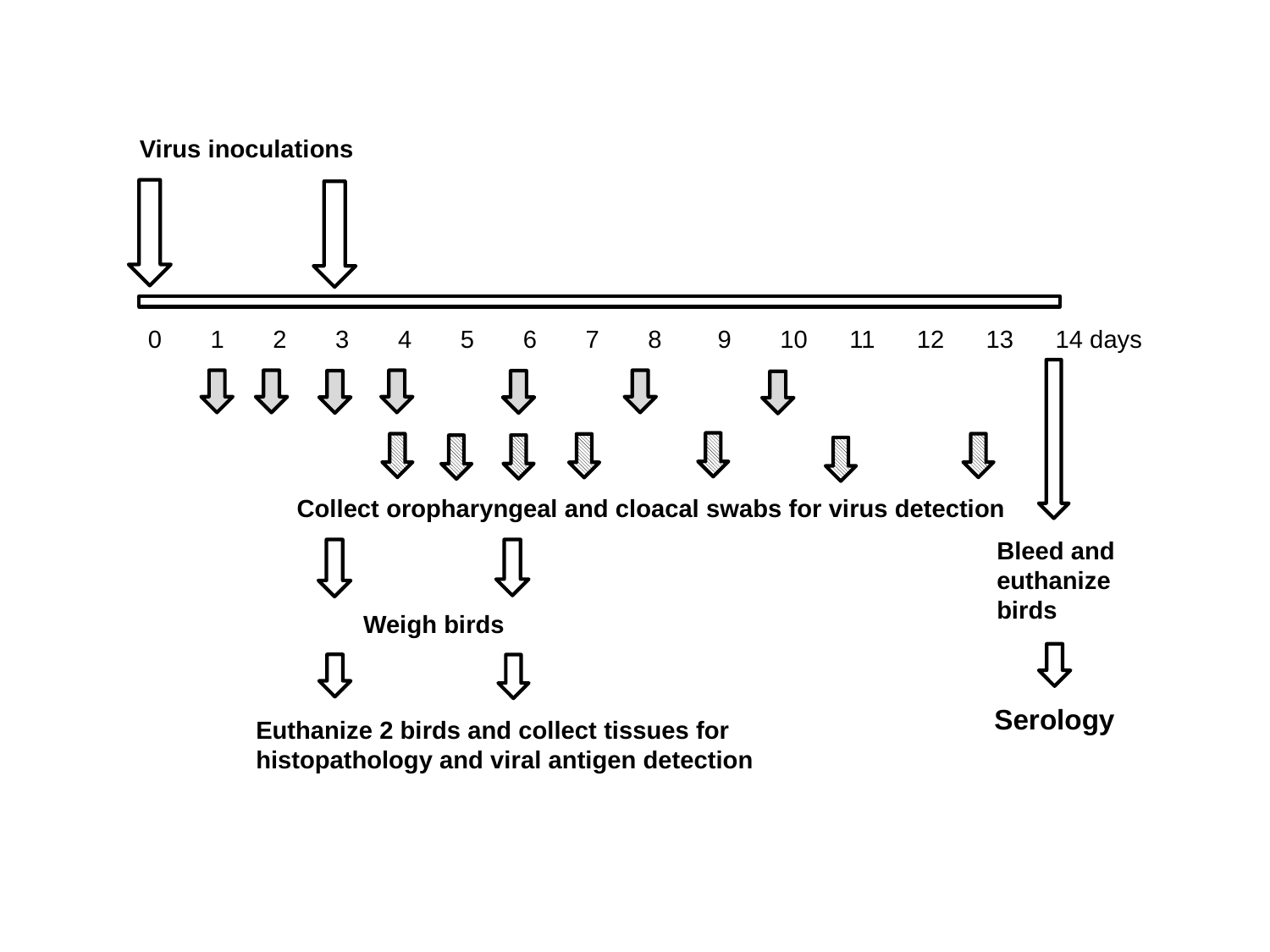

Virus inoculations
0 1 2 3 4 5 6 7 8 9 10 11 12 13 14 days
Collect oropharyngeal and cloacal swabs for virus detection
Bleed and euthanize birds
Weigh birds
Serology
Euthanize 2 birds and collect tissues for histopathology and viral antigen detection
