# Supplementary figures and images for: Virus interference between H7N2 low pathogenic avian influenza virus and lentogenic Newcastle disease virus in experimental co-infections in chickens and turkeys
Source: Vet Res. 2014 Jan 6;45(1):1. doi: 10.1186/1297-9716-45-1 (PMC3890543; doi:10.1186/1297-9716-45-1)

## Slide 1
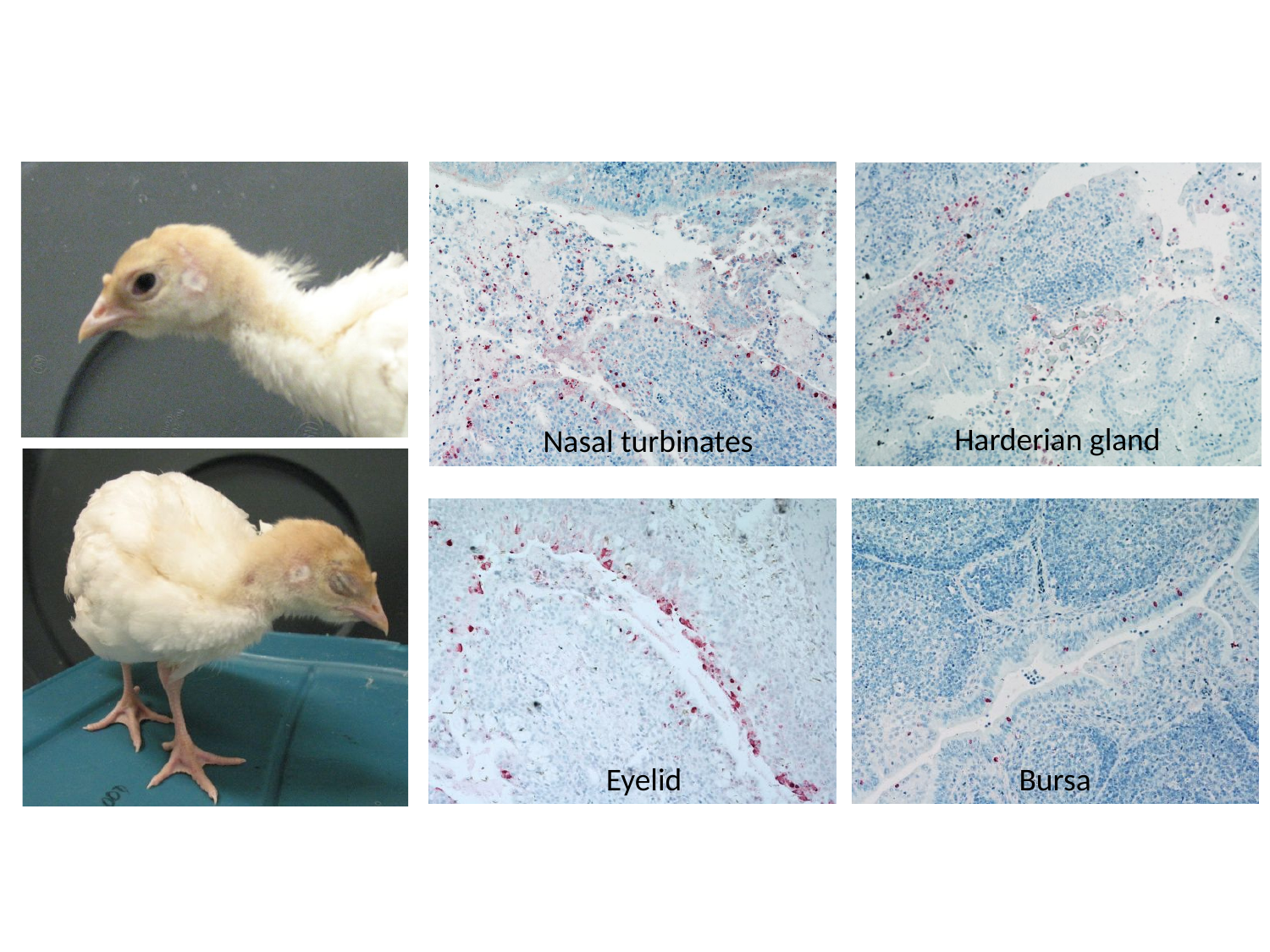

Harderian gland
Nasal turbinates
Eyelid
Bursa

Supplement: Additional file 2 — Clinical signs and distribution of AI viral antigen in tissues collected from turkeys infected with LPAIV, 3 dpi. Infraorbital swelling and conjunctivitis in affected birds. Viral antigen staining (in red) in the epithelium, desquamated cells and infiltrating inflammatory cells of the nasal turbinates, Harderian gland and eyelid, and in the bursa epithelium. [file 1297-9716-45-1-S2.pptx]
